# Piecewise Mixed Effects Model to Compare the Weight-gain Patter ns Before and After Diagnosis of Asthma in Children Younger than 5 Years

**DOI:** 10.4172/2155-6180.1000248

**Published:** 2015-01

**Authors:** Md Jobayer Hossain, Li Xie, Jason E Lang, Timothy T Wysocki, Thomas H Shaffer, H Timothy Bunnell

**Affiliations:** 1 Department of Biomedical Research, Nemours/Alfred I. duPont Hospital for Children, Wilmington, DE, USA; 2 Department of Applied Economics and Statistics, University of Delaware, Newark, DE, USA; 3 Division of Pulmonary and Sleep Medicine, Nemours Children’s Hospital, Orlando, FL, USA; 4 Department of Biomedical Research, Nemours Children's Clinic, Jacksonville, FL, USA; 5 Center for Pediatric Lung Research, Nemours/Alfred I. duPont Hospital for Children, Wilmington, DE, USA

**Keywords:** BMI, Weight-for-length, Piecewise, Mixed effects, Asthma, Weight gain

## Abstract

Asthma and obesity are two significant public health problems that both originate in early childhood and have shared risk factors and manifestations. Studies suggest a strong association between asthma development and subsequent accelerated weight gain. Children are diagnosed with asthma in early childhood and are often exposed to factors associated with rapid weight gain. This article intends to demonstrate an innovative application of the piecewise mixed effects model to characterize the difference in the temporal rate of change in BMIz, the standardized scores of body mass index and weight-for-length that measure weight status, before and after asthma diagnosis in children younger than 5 years. The data consist of unique sequences from 1194 children's clinic visits during the first 5 years of life. We used a knot at the time of diagnosis and detected a differential weight-gain pattern before and after asthma diagnosis. The pre- and post-asthma-diagnosis weight-gain patterns further differ by sex and race-ethnicity. After asthma diagnosis, female children showed a higher increase in the rate of change in BMIz than males. Non-Hispanic African Americans and Hispanics had higher post-diagnosis rates of change in BMIz than Caucasians. The differential weight-gain patterns between male and female children were mainly contributed by Caucasian children. These findings could have important implications in the clinical care of children after asthma diagnosis.

## Introduction

Childhood asthma and child obesity are significant public health problems [1,2]. Asthma in childhood is associated with considerable morbidity and reduced quality of life and is a serious health and economic concern in the United States and worldwide [3,4]. It is the number one cause of hospitalizations in children and the most common chronic condition for days lost from school [4,5]. Although asthma is a chronic, often lifelong disease affecting people of all ages, it onsets primarily in early childhood [4,6]. Fifty percent of all male asthma patients are diagnosed by age three years, and the same percentage of all female cases are diagnosed by age eight years [7]. The prevalence of childhood asthma in the United States was 9.5% in 2011, which was varied to a great extent by age, sex, and race-ethnicity [8].The prevalence is higher in boys before puberty and in girls after puberty, and it is almost double in African Americans (AA) compared to Caucasians for all ages [4]. The lowest prevalence is in Mexican Hispanics [8]. In children younger than 5 years, the prevalence was 6.2% in 2011-7.8% in males and 4.4% in females [8]. In these very young children, the prevalence of asthma in boys and girls by races are 6.1% and 3.2% in Caucasians, 15.1% and 9.4% in AAs, 5.7% and 3.4% in Hispanics (Mexicans), and 17.1% and 10.2% in Hispanics (Puerto Ricans).

Child obesity is another serious health concern and has been identified as an area of needed focus in order to improve the nation’s health [9,10]. Child obesity is defined by body mass index (BMI) for children aged two years or older and by weight-for-length for children younger than two years, and it is associated with reduced quality of life and excess risk for several chronic diseases [11]. BMI and weight-for-length measure the weight relative to height of an individual. According to Centers for Disease Control and Prevention (CDC), children with ≥ 95^th^ percentile of these two measures are obese and within 85^th^ to 95^th^ percentiles are overweight [11]. The proportion of overweight and obese children younger than 18 years has tripled over the last few decades. In 2009–2010, 9.7% of infants and toddlers of aged 0–2 years had a high weight-for-length and 26.7% of children in the United States aged 2–5 years were obese or overweight [11].

A large number of studies have shown a strong association between obesity and asthma but the intrinsic mechanism of this relationship remains largely unknown [12–29]. The impact of this association is much stronger in females than in males [13–17]. It has been long speculated that both asthma and obesity might have common origins [18]. Shared risk factors of these two conditions, such as common genetic markers, aberrant somatic growth, socio-economic status, pre-term birth, smoking during pregnancy, and female sex hormones, are possible mechanisms [18–22]. However, most clinical and epidemiological research on the association of childhood asthma and obesity has focused on a unidirectional relationship with the speculation that obesity leads to an increased risk in the development of asthma [18–23]. While it is challenging to ascertain the causality of the dynamic association of childhood asthma and obesity based on the cross sectional data, the majority of this research relies on the cross sectionally designed studies [23]. Longitudinal studies of this directionality did not account for the possibility of an alternative causal pathway [23]. The potential mechanisms that may explain this directional association include obesity-related increased airway hyper-responsiveness, immune system modification, narrowed airway as a result of chest restriction, and a common genetic susceptibility [23,24]. In contrast to the prevailing hypothesis, a few studies have shown that asthma leads to an increased risk of obesity, and these groups of researchers suggest a bidirectional association of asthma and obesity [23,25–29]. It is plausible that asthma may increase the risk for obesity due to exposure to asthma medications or exercise-avoidance in an attempt to reduce asthma symptoms [13,23,25]. Weight gain is one of the well-known adverse events of oral steroids [13]. Inhaled corticosteroids were found to be associated with weight gain in females and weight loss in males in an adult study [13]. A recent article showed an association of asthma and subsequent weight gain using data from a nationally representative longitudinal study that followed children from kindergarten to middle school [23]. While as many as 50–80 percent of children who have asthma develop symptoms before their fifth birthday and the most economic and health burden is involved with asthma of these young children, our literature search did not yield a longitudinal study concentrating on the association of asthma development and subsequent weight-gain pattern in this group of very young children [6]. The onset and the diagnosis of asthma are two potentially critical time points in the evaluation of the association of the development of asthma and the change in subsequent weight-gain patterns as the mechanisms of this directional association potentially originate at these two time points. Mechanisms related to the onset of asthma could include intrinsic disease or biological markers, and it is difficult to identify the exact time of the onset. While mechanisms related to diagnosis of asthma could include asthma medications or asthma-induced sedentary lifestyles [13,23], in the literature, mechanisms related to the diagnosis are more pronounced [23,25–29]; thus, in this article we used the time of the diagnosis of asthma as the break point for characterizing the association of asthma development and subsequent weight-gain patterns. With a point of time of diagnosis, consideration of a comparative study may not be plausible because of the lack of a suitable control group. A control group of non-asthmatic patients is limited by not having a time point of diagnosis. A study with such a control group only allows comparing the weight-gain pattern between two groups of children over the follow-up period, which is not compatible with our study aim. Moreover, children are diagnosed with asthma at different ages, so even if a contemporary control cohort of the same age group is followed up for the same period of time, it still is not possible to assign an arbitrary time point for the subject in a control group. In the absence of a control group, the optimal method of analysis can be setting a knot at the diagnosis of asthma and then comparing the weight-gain patterns before and after diagnosis. In this article, we intend to demonstrate an innovative application of the piecewise mixed effects model to characterize the difference in the temporal rate of change in BMIz (standardized scores of BMI and weight-for-length) before and after diagnosis of asthma in children younger than 5 years using the data from clinic visits during the first 5 years of life. We used a piecewise mixed effects model with a knot at the diagnosis of asthma for the characterization of individual-patient as well as population-level difference in the temporal trend of BMIz before and after the diagnosis of asthma [30,31]. The difference in the temporal change in BMIz was characterized for overall asthmatic patients, as well as for sex, race-ethnicity, and sex within race-ethnicity. The findings of this study could provide clinicians with valuable insight regarding clinical care for children recently diagnosed with asthma.

## Methods

The study was approved by the Nemours Institutional Review Board (IRB). This is a retrospective, multi-clinic, longitudinal cohort study in which subjects were followed-up from birth to 5 years through their primary care clinic visits. Study subjects were the patients of the Nemours Delaware Valley outpatient clinics who had physician-diagnosed asthma between ages 1 month and less than 5 years.

### Enrollment criteria

Study subjects included 1194 zero- to five-year-old children with physician-diagnosed asthma, who were born between 2001 and 2005 and had their first visit at one of the Nemours Delaware Valley clinics within the first month of birth and at least one clinic visit each year for the next five years of life. Children with medical diagnoses such as cancer, cystic fibrosis, renal failure, and Crohn’s disease were excluded from the study because of the speculation of poor growth. In addition, children without sufficient record of anthropometric measures such as height and weight for the visit criteria listed above were excluded from the study.

### BMI and weight-for-length as the measures of weight status

BMI and weight-for-length are commonly used, reliable methods of measuring body fat and estimating the ideal weight of a person based on height and weight [32]. BMI is calculated as weight/height^2^ (kg/m^2^) for children aged at least 2 years and is not defined for younger children. Weight-for-length is calculated as the average weight (kg) for a given height (m) for children 2 years or younger. BMI-z score is an age- and gender-adjusted standardized score of BMI, and weight-for-length z-score is a gender-adjusted standardized score of weight-for-length. In this paper, we term both standardized measures as BMIz. In an ideal situation, 50% of children are expected to have a BMIz greater than 0, and the rest should have a BMIz less than 0. The BMIz and its percentile are the most commonly used indicators to assess the size and growth patterns of individual children in the United States.

### Study variables and data collection

Data were collected on children’s clinic visits from the first visit to one visit past age 5 years. The clinic-visit data were retrieved retrospectively from the Nemours electronic medical record (EMR) on all patients who met the enrollment criteria.

### Demographic data

Date of birth, age at each visit, sex, race, and ethnicity were collected from the Nemours EMR. To isolate Hispanics from others, we created the variable *race-ethnicity*, combining the information of variables race and ethnicity. This new variable was coded as non-Hispanic Caucasian (Caucasian); non-Hispanic African American (AA); Hispanic Caucasian and Hispanic African American (Hispanics); and other races or missing/refused to disclose races (Others). Age at each visit was calculated in months and years.

### Anthropometric data

Height (m) and weight (kg) data were collected as continuous variables. BMI and weight-for-length were also calculated using collected height and weight data. Standardized scores of BMI and weight-for-length z-score were calculated using CDC SAS code and reference data. As mentioned before, the two standardized scores were named as BMIz in this paper. For data analysis, we used BMIz at the age of 1 month (30 days) as the first measurement and BMIz at the age of 5 years as the last measurement. If the measurements were available for the corresponding ages, we used the values of actual measurements; otherwise, we interpolated BMIz for these two ages using a LOESS (local estimation of scatterplot smoothing) smoother of each child’s own BMIz over time.

### Asthma status

History of asthma was defined as the presence of physician diagnosis of asthma, identified by ICD-9 code 493. Age at the first time of diagnosis of asthma was recorded for each patient.

### Comorbidities

The physician diagnosis data of atopic dermatitis, allergic rhinitis, esophageal reflux, and chronic allergic conjunctivitis were collected.

### Weight gain and weight loss

A positive rate of change in BMIz over time was defined as weight gain, and a negative rate of change in BMIz was defined as weight loss.

## Data Analysis and Results

Data were summarized in tabular and graphical forms. Demographic, anthropometric, and clinical data were summarized by sex, race-ethnicity, and overall patients (Table 1). Quantitative variables were summarized using means and standard errors of means (SE). Categorical variables were summarized using frequencies and percentages. Model assumptions were checked, and appropriate measures were taken in case of violations. The asthma and obesity comorbidities atopy, allergic rhinitis (AR), chronic conjunctivitis, and esophageal reflux were examined as potential confounders of the association of asthma diagnosis and weight-gain pattern. AR and atopy were found to be significantly associated with the change in BMIz and were used in the model adjustment. The Benjamini-Hochberg method was used to adjust for the level of significance for testing 12 differential weight-gain patterns before and after diagnosis of asthma (Table 2). The corrected level of significance was 0.0063 for each test. The statistical software SAS version 9.3 (Cary, NC) was used for the data analysis. All tests were two-sided with an overall level of significance of 0.05.

### Patient characteristics

There were 1194 physician-diagnosed asthma patients who were diagnosed prior to 5 years of age. Table 1 presents the patient characteristics by sex and race-ethnicity. There were 508 (42.55%) female and 686 (57.45%) male children. By race-ethnicity, there were 629 (52.68%) AAs, 354 (29.65%) Caucasians, 163 (13.65%) Hispanics, and 48 (4.02%) others. The mean (SE) age at diagnosis was 1.9 (0.04) years with a median (min-max) of 1.51 (0.08–4.99) years. Male children had a significantly lower mean (SE) age (years) at diagnosis, 1.8 (0.05), compared to that of female children, 2.01 (0.06). Caucasian children had a higher mean (SE) age at diagnosis (years), 2.04 (0.07), than that of AA, 1.82 (0.05), and Hispanic children, 1.80 (0.10). A total of 20359 unique visits were made by 1194 patients, of which 8574 (42.11%) were before diagnosis and 11785 (57.89%) were after diagnosis. There was no substantial difference in the mean number of visits between male and female children in pre- or post-diagnosis periods; however, Caucasian children had a higher mean number of visits than AA and Hispanic children in both pre- and post-diagnosis periods. Atopy, AR, chronic conjunctivitis, and esophageal reflux showed no substantial distributional differences between males and females.

### Visual inspection and model consideration

**Figure 1:**
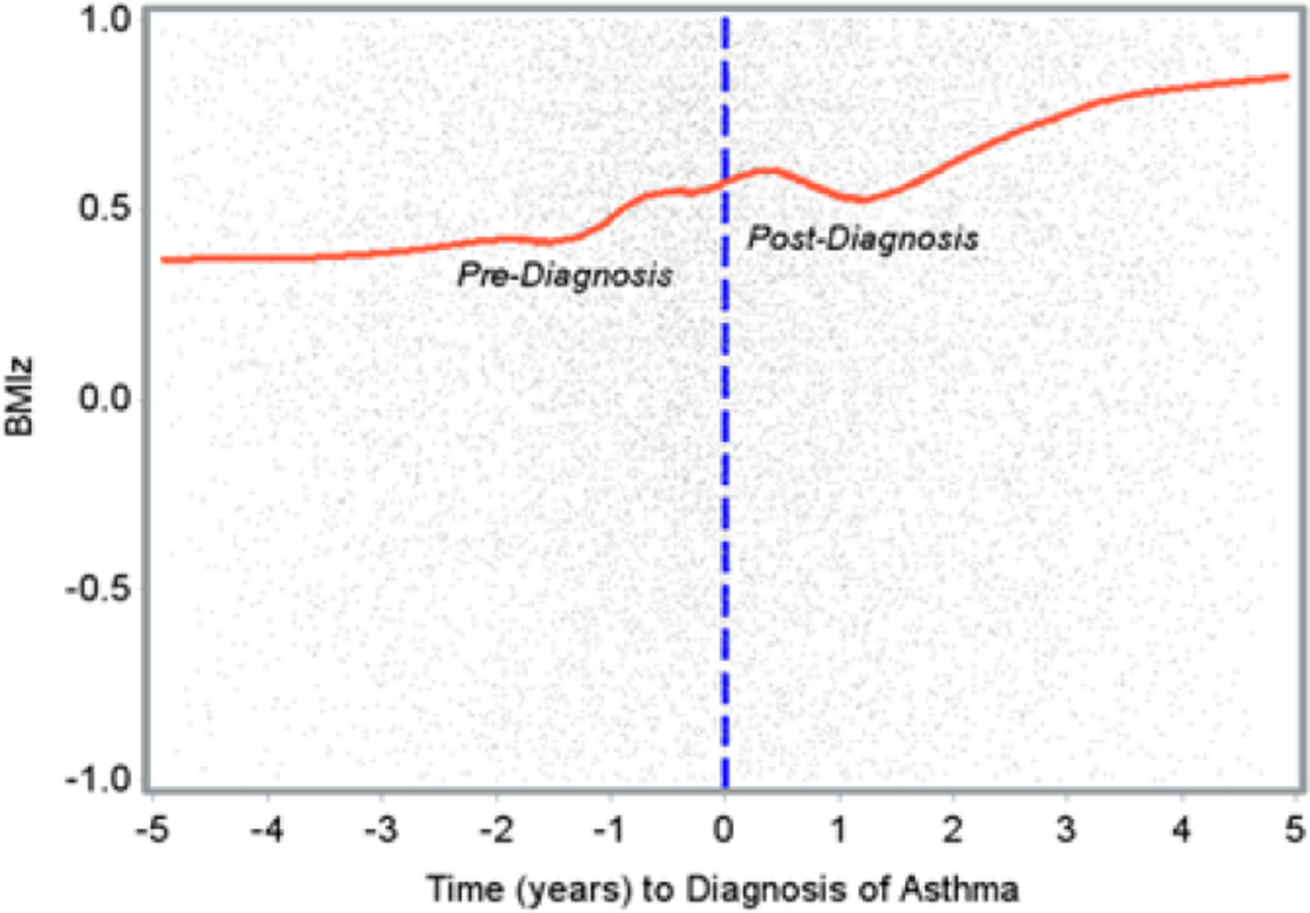
Change in BMlz (Loess curve) before and after diagnosis of asthma.

The LOESS smooth curve in Figure 1 traces the salient feature of the change in BMIz as a function of time. It reveals an approximately linear increase in the rate of change in BMIz over time but, apparently, with different slopes before and after the diagnosis of asthma. There is a slight bump in the rate of change in BMIz around the diagnosis of asthma, and it is difficult to discern the extent of the difference in the rates of changes in BMIz before and after diagnosis from the graph. Analysis with an appropriate model might better expose a difference in the rate of change in BMIz before and after diagnosis of asthma. From the visual inspection of the LOESS curve, we assume that each child has a piecewise linear spline growth curve in the temporal change in BMIz with a knot at the diagnosis of asthma and that we need a model that describes each child’s growth curve with an intercept and two slopes for the trend in the changes in BMIz before and after diagnosis. The collected data were longitudinally completely unbalanced as each subject had a unique sequence of clinic visits over time (range, 6–76 visits). A mixed effects model with random coefficients is appealing in the analysis of this dataset, as the covariance among within-subject repeated measures of BMIz can be expressed as a function of time with relatively fewer parameters, regardless of the number and timing of the clinic visits. We modeled the mean change in BMIz as a combination of population parameters (**β**) that are shared by all individuals, and subject-specific characteristics (***b_i_***) that are unique to each particular individual. For modeling subject-level changes in BMIz, we allowed corresponding regression parameters to vary randomly from one individual to another, and these random regression coefficients account for the individual-level heterogeneity in the population. The following piecewise linear mixed-effect model [33] in equation (1) describes the population- and individual-level change in BMIz before and after diagnosis of asthma in overall population:

(1) $$E(Y_{ij}/b_{i})=\beta_{1}+\beta_{2}t_{ij}+\beta_{3}t_{ij}^{\prime }+b_{1i}+b_{2i}t_{ij}+b_{3i}t_{ij}^{\prime }$$

where *Y_ij_* is the BMIz of the *i^th^* subject at the *j^th^* visit, and *E*(*Y_ij_*/*b_i_*) is the mean BMIz. The variable *t_ij_* denotes the time (since the diagnosis of asthma) of the *j^th^* measurement on the *i^th^* subject before and after diagnosis of asthma with *t_ij_*=0 at the diagnosis of asthma, $t_{ij}^{\prime }=t_{ij}$ if *t_i_* >0 and $t_{ij}^{\prime }=0$ if *t_ij_* ≤ 0. **β**’s are fixed effects that characterize population parameters and ***b_i_***’s are random regression coefficients for individual-level characteristics. Population-level parameters are as follows: β_1_ is the estimated mean (intercept) at diagnosis (i.e., at *t_ij_*=0); β_2_ is the rate of change in BMIz before diagnosis; and β_3_ is the difference in the rate of change in BMIz before and after diagnosis. (β_2_+β_3_) is the rate of change in BMIz after diagnosis. The corresponding subject-level characteristics for *i^th^* subject are β_1_+*b_1i_*, β_2_+*b_2i_*, β_3_+*b_3i_*, and β_2_+β_3_*+b_2i_*+*b_3i_*, respectively. Equation (1) is the basic model that describes the temporal change in BMIz before and after diagnosis of asthma. The age at diagnosis of asthma was different for each patient and was used in the model to account for the cross-sectional effect of this variable. In addition, atopy and allergic rhinitis (AR) were used in the model.

(2) $$E(Y_{ij}/b_{i})=\beta_{1}+\beta_{2}t_{ij}+\beta_{3}t_{ij}^{\prime }+\beta_{4}\text{age}D_{i}+\beta_{5}{\text{Atopy}}_{i}+\beta_{6}AR_{i}+b_{1i}+b_{2i}t_{ij}+b_{3i}t_{ij}^{\prime }$$

In equation (2), *age D_i_* is the age of the diagnosis of asthma of the *i^th^* subject, and *Atopy_i_* and *AR_i_* indicate the presence or absence of atopy and AR of the *i^th^* subject, respectively. The estimates of β_1_, β_2_, β_3_, and β_2_+β_3_ are presented in Table 2 in the row for overall population. In the population-level characteristics, we are mainly interested in the inferences about β_3_, as the main focus of this study is to determine the differences between the slope before and after diagnosis of asthma. There was substantial variability in each of the random coefficients *b_1i_, b_2i_*, and *b_3i_, P*<0.0001. In addition, the expected proportion of subjects to gain or lose weight before and after diagnosis of asthma, and the expected ranges of slopes (before and after diagnosis of asthma) that the 95% subjects lie in, are presented in Table 3 in the row of overall population. Because of a bump around the diagnosis time in Figure 1, we allowed a quadratic trend in the post-asthma-diagnosis period and fit the following model:

(3) $$E(Y_{ij}/b_{i})=\beta_{1}+\beta_{2}t_{ij}+\beta_{3}t_{ij}^{\prime }+\beta_{4}{\left(t_{ij}^{\prime }\right)}^{2}+\beta_{5}\text{age}D_{i}+\beta_{6}{\text{Atopy}}_{i}+\beta_{7}AR_{i}+b_{1i}+b_{2i}t_{ij}+b_{3i}t_{ij}^{\prime }+b_{4i}{\left(t_{ij}^{\prime }\right)}^{2}$$

The inclusion of the quadratic trend in the post-diagnosis period did not lead to a substantial improvement in the model fit of the mean BMIz, *P*=0.08 for the Wald test of β_5_. Based on the Wald test and the residual diagnostics for assessing the goodness of fit, the model in equation (2) is adequate for characterizing the mean BMIz before and after diagnosis.

To characterize the pre- and post-asthma-diagnosis weight-gain patterns in males and females, we added the variable sex and its interaction with time variables *t_ij_* and $t_{ij}^{\prime }$ to equation (2).

(4) $$E(Y_{ij}/b_{i})=\beta_{1}+\beta_{2}{\text{Sex}}_{i}+\beta_{3}t_{ij}+\beta_{4}t_{ij}^{\prime }+\beta_{5}t_{ij}\times {\text{Sex}}_{i}+\beta_{6}t_{ij}^{\prime }\times {\text{Sex}}_{i}\;+\beta_{4}\text{age}D_{i}+\beta_{5}{\text{Atopy}}_{i}+\beta_{6}AR_{i}+b_{1i}+b_{2i}t_{ij}+b_{3i}t_{ij}^{\prime }$$

where *Sex_i_* =1 if the *i*^th^ patient is a male, and *Sex_i_=0* otherwise. Table 2 presents the sex-specific characterization for female and the difference of the male and female.

For race-ethnicity specific characterization, we created three indicators for AA, Hispanics, and others. Caucasian was the reference group. Again, we used these three indicators and their interactions with time variables *t_ij_* and $t_{ij}^{\prime }$ in the equation. Table 2 presents the race-ethnicity characterization for Caucasians (reference) and its difference with AAs and Hispanics.

Finally, we applied the equation (4) to the data of each of the race-ethnicity to characterize the pre- and post-asthma-diagnosis weight-gain patterns in males and females within each race-ethnicity. Table 2 and 3 present the estimates of all models discussed above.

### Differential weight gain before and after diagnosis of asthma in children overall

The mean (SE) BMIz at diagnosis of asthma was 0.489 (0.054). There was a substantially higher rate of change in BMIz during the post-asthma-diagnosis compared to the pre-asthma-diagnosis period. The difference in the yearly rate (SE) of change in BMIz between pre- and post-asthma diagnosis was 0.077 (0.023), *P*=0.0009. There was a trivial (yearly) change in BMIz before diagnosis of asthma with a rather shallow slope (SE)=−0.0038 (0.0192), *P*=0.8418. However, there was a sharp increasing trend in BMIz after diagnosis of asthma with a slope (SE) of 0.0730 (0.0097), *P*<0.0001. There was significant variability in the individual-level rate of change during pre- and post-asthma diagnosis periods as well as in the difference of the rate of change in BMIz before and after diagnosis, *P*<0.0001. Approximately 95% children had a yearly rate of change in BMIz between −1.07 and 1.06 before diagnosis and between −0.47 and 0.62 after diagnosis of asthma. This indicated that there was a greater variability in the slope before diagnosis of asthma and that not all children gained or lost weight during both pre- and post-diagnosis of asthma; rather, some gained and some lost weight. Approximately 61.36% of children were expected to have increases in BMIz after diagnosis, while only 49.7% of children were expected to have increases before diagnosis of asthma.

### Differential weight gain in male and female children before and after asthma diagnosis

The estimated mean BMIz at diagnosis of asthma was higher in males than in females. This estimated mean (SE) was 0.357 (0.055), *P*<0.0001, in female children and the difference in mean (SE) BMIz between males and females was 0.160 (0.064), *P*=0.0114. There was a substantial difference in the estimated rate (SE) of change in BMIz before and after diagnosis of asthma in females, 0.134 (0.035), *P*=0.0001. This difference indicates a significant shift with a sharp increasing trend in the change in BMIz after diagnosis of asthma in the female children. The trend of the change in BMIz in females showed a trivial decrease before diagnosis, with a shallow slope (SE) of −0.047 (0.029), *P*=0.0985, and a rapid increase after diagnosis, with a steeper slope (SE) of 0.087 (0.015), *P*<0.0001. Compared to females, males showed a substantially higher increase in the trend of the change in BMIz before diagnosis, while a lower increase after diagnosis of asthma. The corresponding estimated differences (SE) in slopes between males and females are 0.082 (0.038), *P*=0.0311, and −0.023 (0.020), *P*=0.2474, respectively. The contrasting features of the changes in BMIz between males and females generated a wide difference between these two groups of children in terms of the difference in the trend of the change in BMIz before and after diagnosis of asthma. The estimated difference (SE) of this parameter between male and female was −0.105 (0.046), *P*=0.0228. Approximately 95% female children were expected to have a yearly rate of change in BMIz between −1.02 and 0.91 before diagnosis and between −0.40 and 0.57 after diagnosis of asthma, while in males the corresponding intervals were −1.11 to 1.05 and −0.52 to 0.65, respectively. About 45.92% and 63.21% of females and 52.33% and 58.60% of males were expected to have an increasing BMIz before and after diagnosis of asthma, respectively.

### Differential race-ethnicity-specific weight gain before and after asthma diagnosis

The estimated mean (SE) BMIz at diagnosis in Caucasian children was 0.509 (0.075), *P*<0.0001. This estimated mean was slightly lower in AAs and higher in Hispanics than in Caucasians. The difference (SE) in mean BMIz between AAs and Caucasians was −0.092 (0.073), *P*=0.2091, and between Hispanics and Caucasians was 0.154 (0.104), *P*=0.1401, but these differences were not even significant at the level of significance of 0.05. Caucasian children had an increasing trend in the change in BMIz both before and after diagnosis of asthma, but with a steeper slope before diagnosis. The corresponding estimated slopes (SE) were 0.092 (0.034), *P*=0.0066, and 0.068 (0.018), *P*=0.0002, before and after diagnosis of asthma, respectively. The lower variability in individual-level post-diagnosis slopes yielded a lower *P*-value even with a shallower slope than pre-diagnosis. The difference (SE) between the estimated slopes before and after diagnosis was −0.028 (0.041), *P*=0.554 in Caucasian children. Compared to them, AA and Hispanic children had a substantially smaller estimated slope of the temporal change in BMIz before diagnosis of asthma. The corresponding estimated difference in slope (SE) between Caucasians and AAs was −0.145(0.043), *P*=0.0007, and between Caucasians and Hispanics was −0.129 (0.062), *P*=0.0355. There was a trivial difference in the magnitude of the temporal change in BMIz after diagnosis of asthma among these three race-ethnicity groups. It indicated that, like in Caucasians, there was a substantial increasing trend in the change in BMIz after diagnosis of asthma in AA and Hispanic children; however, the difference (SE) in the rate of change in BMIz between pre- and post-diagnosis was substantially larger in AAs and in Hispanics than in Caucasians. The estimated difference of the corresponding slope (SE) was 0.152 (0.052), *P*=0.0032, between Caucasians and AAs and 0.137 (0.073), *P*=0.0644, between Caucasians and Hispanics. Approximately 95% of Caucasian children were expected to have a yearly rate of change in BMIz between −0.90 and 1.10 before diagnosis and between −0.56 and 0.70 after diagnosis of asthma. In AA children, these intervals were −1.15 to 1.04 and −0.45 to 0.59, respectively and in Hispanics were −1.25 to 1.58 and −0.45 to 0.60, respectively. Hispanics showed a relatively higher variability in the rate of change in BMIz before diagnosis, and Caucasians showed a higher variability after diagnosis of asthma. About 57.57% and 58.56% of Caucasian children, 45.83% and 60.77% of AA children, and 47.03% and 61.40% of Hispanic children were expected to have an increasing BMIz before and after diagnosis of asthma, respectively.

### Differential weight gain in male and female children within race-ethnicity

In Caucasian female children, the estimated mean (SE) BMIz at diagnosis was 0.192 (0.127), *P*=0.1314; the estimated rate (SE) change in BMIz before diagnosis was −0.036 (0.050), *P*=0.4718; the rate (SE) of change in BMIz after diagnosis was 0.118 (0.033), *P*=0.0004; and the difference in the rate (SE) of change in BMIz was 0.153 (0.063), *P*=0.016. Compared to Caucasian females, males had a higher mean (SE) of BMIz at diagnosis, 0.385 (0.116), *P*=0.0009; higher rate (SE) of the change in BMIz before diagnosis, 0.225 (0.064), *P*=0.0005; and a lower rate of the change in BMIz after diagnosis of asthma, −0.078 (0.041), *P*=0.0584. Male children had a substantially steeper rate in the change in BMIz before diagnosis, while female children had a steeper rate after diagnosis of asthma. These contrasting patterns of the trends in the changes in pre- and post-diagnosis BMIz between Caucasian males and females caused a wide difference between males and females with respect to the difference in the pre- and post-diagnosis of asthma. The estimated difference (SE) between males and females of this parameter (β_3_) was −0.303 (0.082), *P*=0.0002. Caucasian females had less variability in the rate of change in BMIz before and after diagnosis of asthma compared to the Caucasian males. Approximately 46.77% of Caucasian females and 62.34% of Caucasian males are expected to have an increasing trend in the BMIz before diagnosis, while after diagnosis, 66.64% of females and 54.14% of males are expected to have an increasing trend.

In contrast to Caucasians, AA and Hispanic children did not show a substantial difference between males and females with respect to the estimates of the four parameters of our interest. The estimated mean (SE) BMIz at the diagnosis of asthma in AA and Hispanic females was 0.394 (0.089) and 0.804 (0.186), respectively. The estimated difference in the rate (SE) of change in BMIz before and after diagnosis was 0.149 (0.049), *P*=0.0024, and 0.132 (0.098), *P*=0.1793, in these two groups, respectively, indicating a substantial increase in the rate of change in BMIz after diagnosis of asthma in these children. The pre-diagnosis rates of change in BMIz were not substantially different in females of all three race-ethnicities, with a trivial decreasing trend in the change in BMIz. In AA children, 44.60% females and 46.68% males were expected to have an increasing trend in the BMIz before diagnosis, and after diagnosis, 61.92% females and 60.03% males were expected to have an increasing trend. In Hispanic children, about 44.23% females and 48.05% males were expected to have an increasing trend before diagnosis of asthma, and 61.07% females and 61.62% males were expected to have an increase in the rate of change in BMIz after diagnosis.

## Discussion

The data of this study were collected from a dataset of children both with and without asthma and had a natural balance of the distribution of male and female children. However, the proportion of boys (57.45%) is higher than girls (42.55%) in this study which is consistent with the existing literature of the prevalence of childhood asthma. The majority of the children in this study are AA (52.68%). In the national data, the prevalence of asthma in AA is almost double that of Caucasian children. The mean age at diagnosis of asthma is higher in female and in Caucasian children compared to their counterparts. The average visit frequency is higher in Caucasians. The prevalence of atopy was significantly higher in AA children, and the prevalence of gastroesophageal reflux was higher in Caucasians.

There was a significant difference in the temporal rate of change in BMIz before and after diagnosis with an increasing rate of weight gain following diagnosis. This difference in the pre-and post-asthma diagnosis weight-gain pattern varied significantly by sex and race-ethnicity even after accounting for the effect of age at diagnosis, atopy, and AR. Although, there was a distributional difference of gastroesophageal reflux over race-ethnicity, this variable had a negligible influence on the model fitness and was dropped from the model. Compared to males, females showed a substantially higher weight gain after the diagnosis of asthma than before diagnosis. This might be attributed to the differential effect of some asthma medications.

There was no significant difference in the temporal rate of change in BMIz before and after diagnosis of asthma in Caucasian children. In fact, female Caucasian children showed a rapid accelerated weight gain after diagnosis, but male children showed significant down trend in the change in BMIz. These contrasting weight-gain patterns between male and female Caucasian children neutralized the difference of the pre- and post-diagnosis rate of change in the combined Caucasian population.

However, there was a significant difference between Caucasians, AAs, and Hispanics in terms of the difference in the trend of the change in BMIz before and after diagnosis of asthma. Both of the latter race-ethnicities showed a significant difference in the change in BMIz before and after diagnosis that is driven by post-diagnosis accelerated weight gain. Unlike Caucasian children, there was no considerable difference between males and females of these two race-ethnicities. The differential post-diagnosis-accelerated weight gain between race-ethnicity may be related to the differences in asthma medication use or other factors, such as socio-economic status of family, or it could even be the biological difference between race-ethnicity groups.

## Limitations

The data were collected on children’s clinic visits and retrospectively retrieved from the EMR. We had a very limited scope to resolve any inconsistencies in the data inherited from the measurement error, but the Nemours EMR system is technologically equipped to minimize such inconsistencies and missing data. For example, the Nemours electronic data entry system prompts a caution if the height for the current visit is less than the height recorded for the past visit. There are also checks for the height and weight measurement units through data entry prompts if the entered height and weight differ more than a certain percentage at different ages. There were no missing sex data in the dataset, and only seven patients had missing information on race-ethnicity.

The positioning of the newborn children during measurement and other factors may cause stochastic fluctuations in both weight and height measures. Even a small measurement error can produce a substantial error in weight-for-length z-score (BMIz), affecting the reliability of the measurement of this variable in very early age. Moreover, children often had frequent visits at a young age because of vaccinations and other monitoring. To avoid using BMIz of frequent visits in very early ages in the analysis, we used BMIz at the age of 1 month (30 days) as the first measurement in the data analysis.

Finally, the detected differential changes in BMIz before and after diagnosis of asthma might be partially driven by the unmeasured aberrant nature of the early-age growth pattern. In the absence of a control group, which is impractical in this setting, it is not possible to account for this confounding effect. However, the positive correlation of asthma onset with subsequent weight gain that was reported in a study of older children supports our findings [23].

## Conclusions

In summary, we used a piecewise mixed effects model with a knot at the diagnosis and detected a differential trend in the temporal change in BMIz before and after diagnosis of asthma while accounting for the effect of age at diagnosis and for comorbidities such as atopy and AR. The weight-gain patterns before and after diagnosis further differ by sex and race-ethnicity. After diagnosis of asthma, female children showed a higher increase in the rate of change in BMIz than males. Similarly, non-Hispanic African Americans (AAs) and Hispanics had higher post-diagnosis rates of change in BMIz than in Caucasians. The differential weight-gain patterns between male and female children were mainly contributed by Caucasian children. Our findings, in concert with previously reported results in relatively older children, suggest that the development of asthma in children may be associated with accelerated weight gain, which eventually leads to the onset of obesity. Future studies could focus on quantifying the potential determinants of the association asthma and accelerated weight gain and childhood obesity.

## Figures and Tables

**Table 1:** Patients characteristics.

Variable	Sex		Overall
	Female	Male	
Number of Subjects (%)*	508 (42.55)	686(57.45)	1194 (100)
**Race-ethnicity, n (%)**			
Caucasian	139 (27.36)	215 (31.34)	354 (29.65)
African American	279 (54.92)	350 (51.02)	629 (52.68)
Hispanic	70 (13.78)	93 (13.56)	163 (13.65)
Others/missing	20 (3.94)	28 (4.08)	48 (4.02)
**Visit frequencies**			
Number of visits	8574	11785	20359
Mean (SE)	16.88 (0.32)	17.18 (0.28)	17.05 (0.21)
**Pre-diagnosis visit frequencies**			
Number of visits	4105	5380	9485
Mean (SE)	8.08 (0.22)	7.84 (0.19)	7.94 (0.15)
Post-diagnosis visit frequencies			
Number of visits	4469	6405	10874
Mean (SE)	8.8 (0.28)	9.34 (0.25)	9.11 (0.19)
Age at Diagnosis (Yrs), mean (SE)*	2.01 (0.06)	1.81 (0.05)	1.90 (0.04)
Comorbidities, n (%)			
Atopy	28 (5.51)	42 (6.12)	70 (5.86)
Allergic rhinitis	249 (49.02)	318 (46.36)	567 (47.49)
Chronic conjunctivitis	10 (1.97)	15 (2.19)	25 (2.09)
Gastroesophageal reflux	134 (26.38)	204 (29.74)	338 (28.31)
	**Race-Ethnicity**		
**Variable**	**Caucasian**	**African American**	**Hispanic**
Visit frequencies**			
Number of visits	6867	10052	2660
Mean (SE)	19.40 (0.46)	15.98 (0.26)	16.32 (0.48)
Pre-diagnosis visit frequencies**			
Number of visits	3395	4516	1155
Mean (SE)	9.59 (0.34)	7.18 (0.16)	7.09 (0.30)
Post-diagnosis visit frequencies			
Number of visits	3472	5536	1505
Mean (SE)	9.81 (0.38)	8.80 (0.25)	9.23 (0.46)
Age at Diagnosis (years), mean (SE)*	2.04 (0.07)	1.82 (0.05)	1.80 (0.10)
Comorbidities, n (%)			
Atopy**	14 (3.95)	50 (7.95)	4 (2.95)
Allergic rhinitis	178 (50.28)	281 (44.67)	82 (50.31)
Chronic conjunctivitis	6 (1.69)	13 (2.07)	4 (2.45)
Gastroesophageal reflux**	121 (34.18)	177 (28.14)	29 (17.79)

**Note:**

* 0.05 ≤ *P*<0.01;

** 0.01 ≤ *P*<0.001;

SE, standard error.

**Table 2:** Temporal change in BMIz before and after diagnosis.

Groups	BMIz Estimates							
	At Diagnosis		Pre-Diagnosis		Post-Diagnosis		ΔPost-Pre Diagnosis	
	Mean (SE)	*P*-value	Mean (SE)	*P*-value	Mean (SE)	*P*-value	Mean (SE)	*P*-value
**Overall**	0.489 (0.054)	<0.0001	−0.004 (0.019)	0.8418	0.073 (0.010)	<0.0001	0.077 (0.023)	0.0009
**Sex**								
Female (Ref)	0.357 (0.055)	<0.0001	−0.047 (0.029)	0.0985	0.087 (0.015)	<0.0001	0.134 (0.035)	0.0001
Male* (Diff)	0.160 (0.064)	0.0114	0.082 (0.038)	0.0311	−0.023 (0.020)	0.2474	−0.105 (0.046)	0.0228
**Race-Ethnicity**								
Caucasian (Ref)	0.509 (0.075)	<0.0001	0.092 (0.034)	0.0066	0.068 (0.018)	0.0002	−0.028 (0.041)	0.554
African American* (Diff)	−0.092 (0.073)	0.2091	−0.145 (0.043)	0.0007	0.008 (0.022)	0.7303	0.152 (0.052)	0.0032
Hispanic* (Diff)	0.154 (0.104)	0.1401	−0.129 (0.062)	0.0355	0.008 (0.034)	0.8027	0.137 (0.073)	0.0644
**Sex within Race-Ethnicity**								
Caucasian								
Female (Ref)	0.192 (0.127)	0.1314	−0.036 (0.050)	0.4718	0.118 (0.033)	0.0004	0.153 (0.063)	0.016
Male* (Diff)	0.385 (0.116)	0.0009	0.225 (0.064)	0.0005	−0.078 (0.041)	0.0584	−0.303 (0.082)	0.0002
African American								
Female (Ref)	0.394 (0.089)	<0.0001	−0.073 (0.050)	0.074	0.076 (0.019)	0.0001	0.149 (0.049)	0.0024
Male* (Diff)	0.099 (0.088)	0.2638	0.026 (0.055)	0.6291	−0.006 (0.026)	0.8047	−0.033 (0.065)	0.6162
Hispanic								
Female (Ref)	0.804 (0.186)	<0.0001	−0.062 (0.086)	0.4763	0.070 (0.039)	0.0694	0.132 (0.098)	0.1793
Male* (Diff)	0.005 (0.182)	0.9794	0.27 (0.113)	0.8088	0.013 (0.050)	0.802	−0.015 (0.128)	0.9085

**Note:** Δ, difference; Ref, reference group;

* Diff, difference between corresponding and reference groups.

**Table 3:** Characterization of the individual-level BMIz rate of change before and after asthma diagnosis.

Group	Before Diagnosis		After Diagnosis	
	95% CI of Rate of Change in BMIz	Expected Proportion of Weight Gain (%)	95% CI of Rate of Change in BMIz	Expected Proportion of Weight Gain (%)
**Overall**	−1.07, 1.06	49.70	−0.47, 0.62	61.36
**Sex**				
Female	−1.02, 0.91	45.92	−0.40, 0.57	63.21
Male	−1.11, 1.05	52.33	−0.52, 0.65	58.60
**Race-Ethnicity**				
Caucasian	−0.90, 1.10	57.57	−0.56, 0.70	58.56
African American	−1.15, 1.04	45.83	−0.45, 0.59	60.77
Hispanic	−1.25, 1.58	47.03	−0.45, 0.60	61.40
**Sex within Race-Ethnicity**				
Caucasian				
Female	−0.74, 0.68	46.77	−0.42, 0.65	66.64
Male	−1.05, 1.45	62.34	−0.64, 0.71	54.14
African American				
Female	−1.33, 1.16	44.60	−0.40, 0.55	61.92
Male	−1.06, 0.97	46.68	−0.48, 0.62	60.03
Hispanic				
Female	−1.03, 0.89	44.23	−0.41, 0.54	61.07
Male	−1.41, 1.34	48.05	−0.48, 0.65	61.62
